# Comparisons between cross-section and long-axis-section in the quantification of aneurysmal wall enhancement of fusiform intracranial aneurysms in identifying aneurysmal symptoms

**DOI:** 10.3389/fneur.2022.945526

**Published:** 2022-07-22

**Authors:** Fei Peng, Lang Liu, Hao Niu, Xin Feng, Hong Zhang, Xiaoxin He, Jiaxiang Xia, Boya Xu, Xiaoyan Bai, Zhiye Li, Binbin Sui, Aihua Liu

**Affiliations:** ^1^Department of Interventional Neuroradiology, Beijing Tiantan Hospital, Capital Medical University, Beijing, China; ^2^Department of Neurosurgery, The Third Xiangya Hospital, Central South University, Changsha, China; ^3^Neurosurgery Center, Department of Cerebrovascular Surgery, Engineering Technology Research Center of Education Ministry of China on Diagnosis and Treatment of Cerebrovascular Disease, Zhujiang Hospital, Southern Medical University, Guangzhou, China; ^4^Operating Room, Heze Municipal Hospital, Heze, China; ^5^Department of Radiology, Beijing Tiantan Hospital, Capital Medical University, Beijing, China; ^6^Tiantan Neuroimaging Center of Excellence, China National Clinical Research Center for Neurological Diseases, Beijing, China

**Keywords:** fusiform aneurysm, aneurysm wall enhancement, MRI, symptom, quantification

## Abstract

**Background:**

To investigate the quantification of aneurysmal wall enhancement (AWE) in fusiform intracranial aneurysms (FIAs) and to compare AWE parameters based on different sections of FIAs in identifying aneurysm symptoms.

**Methods:**

Consecutive patients were prospectively recruited from February 2017 to November 2019. Aneurysm-related symptoms were defined as sentinel headache and oculomotor nerve palsy. All patients underwent high resolution magnetic resonance imaging (HR-MRI) protocol, including both pre and post-contrast imaging. CR_stalk_ (signal intensity of FIAs' wall divided by pituitary infundibulum) was evaluated both in the cross-section (CR_stalk−cross_) and the long-axis section (CR_stalk−long_) of FIAs. Aneurysm characteristics include the maximal diameter of the cross-section (*D*_max_), the maximal length of the long-axis section (*L*_max_), location, type, and mural thrombus. The performance of parameters for differentiating symptomatic and asymptomatic FIAs was obtained and compared by a receiver operating characteristic (ROC) curve.

**Results:**

Forty-three FIAs were found in 43 patients. Eighteen (41.9%) patients who presented with aneurysmal symptoms were classified in the symptomatic group. In univariate analysis, male sex (*P* = 0.133), age (*P* = 0.013), FIAs type (*P* = 0.167), mural thrombus (*P* = 0.130), *L*_max_ (*P* = 0.066), CR_stalk−cross_ (*P* = 0.027), and CR_stalk−long_ (*P* = 0.055) tended to be associated with aneurysmal symptoms. In the cross-section model of multivariate analysis, male (*P* = 0.038), age (*P* = 0.018), and CR_stalk−cross_ (*P* = 0.048) were independently associated with aneurysmal symptoms. In the long-axis section model of multivariate analysis, male (*P* = 0.040), age (*P* = 0.010), CR_stalk−long_ (*P* = 0.046), and *L*_max_ (*P* = 0.019) were independently associated with aneurysmal symptoms. In the combination model of multivariate analysis, male (*P* = 0.027), age (*P* = 0.011), CR_stalk−cross_ (*P* = 0.030), and *L*_max_ (*P* = 0.020) were independently associated with aneurysmal symptoms. CR_stalk−cross_ has the highest accuracy in predicting aneurysmal symptoms (AUC = 0.701). The combination of CR_stalk−cross_ and *L*_max_ exhibited the highest performance in discriminating symptomatic from asymptomatic FIAs (AUC = 0.780).

**Conclusion:**

Aneurysmal wall enhancement is associated with symptomatic FIAs. CR_stalk−cross_ and *L*_max_ were independent risk factors for aneurysmal symptoms. The combination of these two factors may improve the predictive performance of aneurysmal symptoms and may also help to stratify the instability of FIAs in future studies.

## Introduction

Intracranial aneurysms (IAs) affect nearly 3%−5% of the whole population (1). IAs can be classified as saccular intracranial aneurysms (SIAs) and fusiform intracranial aneurysms (FIAs) based on aneurysm morphology. Among them, SIAs are the majority (2), while FIAs are an uncommon type, which only stand for 3%−13% of IAs (3–5). Compared with SIAs, FIAs depict different and numerous pathological processes (2, 6, 7). Based on pathological features, FIAs were divided into three types which include fusiform, dolichoectatic, and transitional (8).

It is reported that aneurysm growth and rupture are mediated by inflammation processes (IPs) of the aneurysmal wall (9). Recently, aneurysmal wall enhancement (AWE) in high-resolution magnetic resonance imaging (HR-MRI) has been demonstrated as the biomarker of aneurysmal wall inflammation (10, 11). Aneurysm-to-pituitary stalk (CR_stalk_) was reported to be the most reliable AWE quantitative parameter in SIAs (12). In addition, CR_stalk_ was independently associated with aneurysmal symptoms in SIAs (13, 14). Similar results were also demonstrated in the study of FIAs (15). Notably, the quantification of AWE in SIAs was based on the largest section, which is located at the maximal diameter (7, 16). However, previous studies demonstrated diverse sections for AWE quantification in FIAs, which include the section with the greatest AWE, the largest aneurysmal section, and the section located at the maximal diameter (2, 7, 15). Recent studies revealed the parent vessel manifested higher enhancement in the area close to the neck of SIAs (7). Considering the pathological processes of FIAs may affect the wider involved vessel than SIAs (7), the long-axis section of FIAs which include the dilation and part of the involved artery may represent the overall influence area of FIAs, while the cross-section of the most obvious dilation may represent the degree of progression of FIAs since this section has been proved to be independently associated with aneurysm growth (16). AWE parameters based on these two sections may comprehensively reflect the aneurysmal IPs in FIAs. However, no studies have shown the CR_stalk_ in which the section is more related to aneurysmal symptoms. As it is reported that aneurysmal symptoms (sentinel headache and oculomotor nerve palsy) may reflect the instability of IAs (14), investigating the association between CR_stalk_ and aneurysmal symptoms may help to further understand the potential mechanisms of FIAs' instability.

This study aimed to investigate the quantification of AWE in FIAs based on two different sections and investigate the association between CR_stalk_ in two sections of FIAs and aneurysmal symptoms to distinguish CR_stalk_ in which section has the highest specificity in distinguishing aneurysmal symptoms.

## Methods

### Patient population and data collection

**Figure 1:**
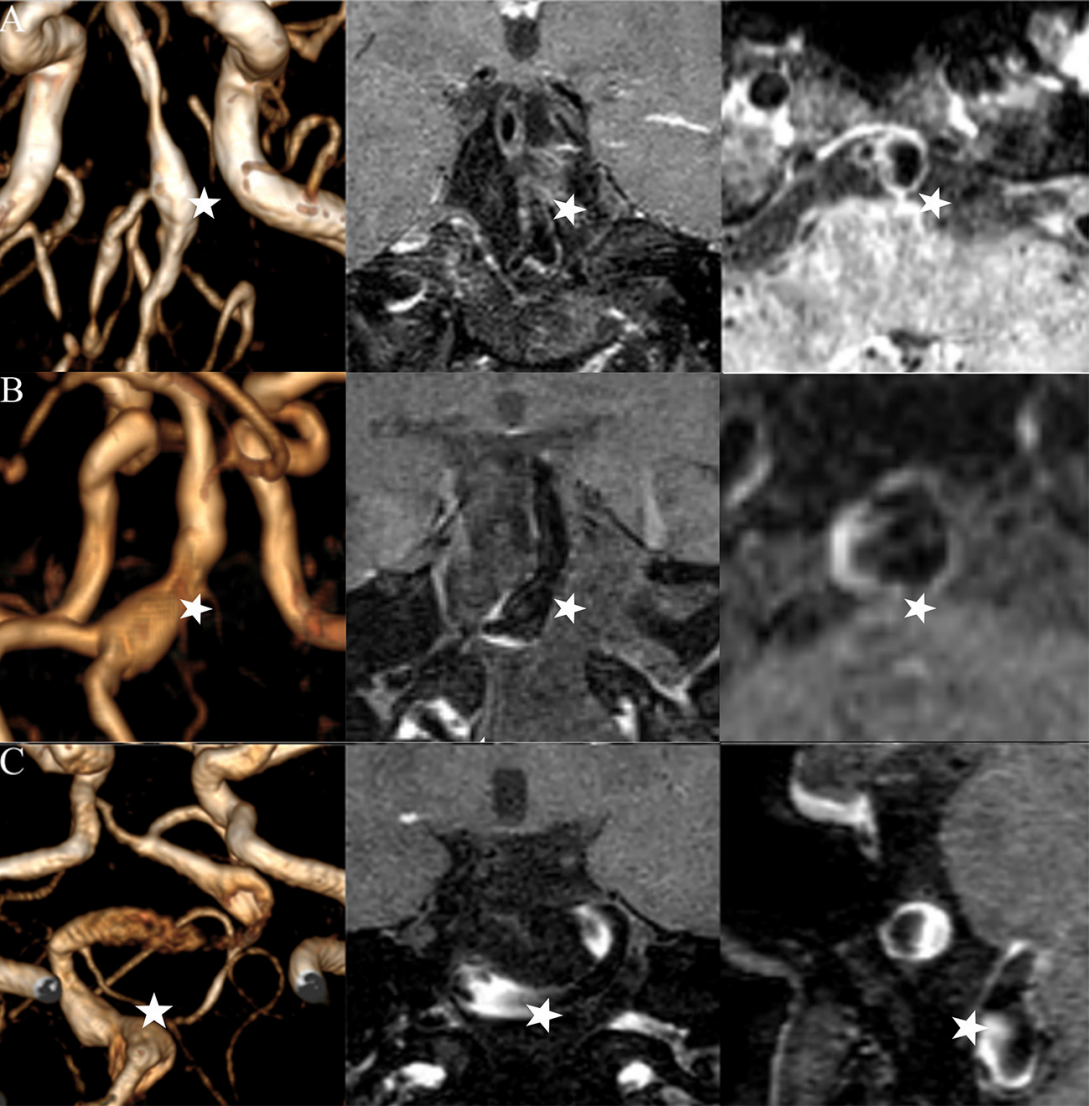
Three types of FIAs: fusiform **(A)**, dolichoectatic **(B)**, and transitional **(C)**. Time-of-flight MR images (left column), post-contrast high-resolution MR images in the long-axis of the FIAs (middle column), and post-contrast high-resolution MR images in the cross-section of the FIAs (right column) are illustrated by each IFA (star).

This prospective study was approved by the local Institutional Review Board of Beijing Tiantan Hospital, and written informed consent was obtained from each patient. The database included consecutive patients with FIAs performed by HR-MRI in Beijing Tiantan Hospital from February 2017 to November 2019. Aneurysmal symptoms were categorized by sentinel headache, oculomotor nerve palsy, and other cranial nerve symptoms (e.g. trigeminal neuralgia). Sentinel headache was defined as a severe, sudden onset headache (14). As sentinel headache and oculomotor nerve palsy may strongly indicate aneurysm instability (14), and these two symptoms were used in the investigation of the association between aneurysmal symptoms and CR_stalk_ in two sections of FIAs. Considering the distinct pathophysiological differences between dissecting aneurysms (featured in double lumen, string sign, or intimal flap, etc.) and other FIAs (15), one experienced reader identified and excluded the dissecting aneurysms. Then, the rest FIAs were defined as three types based on Flemmings' classification: fusiform type, dolichoectatic type, and the transitional type (Figure 1) (8). Mural thrombus was defined as high T1 signal (7). We excluded those patients with poor image quality, incomplete medical records, other saccular or dissecting aneurysms, and FIAs which were related to arteriovenous malformations (AVMs), dural arteriovenous fistulas (DAVFs), and moyamoya disease. Demographics of patients were obtained from electronic medical records.

### Imaging acquisition

All the MRI scans were performed on 3.0T MR scanners (Trio-Tim, Siemens Healthcare, Erlangen; Ingenia CX, Philips Healthcare, Best; Discovery 750, GE Healthcare, Milwaukee, WI) with a 32-channel head coil. Three-dimensional time-of-flight (3D TOF) magnetic resonance angiography (MRA) was used for the localization of the FIAs. The HR-MRI protocol included 3D T1WI (SPACE/VISTA/CUBE), 3D T2/PDWI (SPACE/VISTA/CUBE), and contrast-enhanced 3D T1WI (SPACE/VISTA/CUBE). We acquired the images in the oblique coronal plane, which covers the whole aneurysm. The voxel size was 0.7 × 0.7 × 0.7 mm^3^. Post-contrast T1W images were obtained 6 min after Gd injection (0.1 mmol/kg gadopentetate dimeglumine, Magnevist; Bayer Schering Pharma AG) using parameters identical to those of the pre-contrast T1W images.

### HR-MRI assessment

Quantitative analysis of HR-MRI images was performed with Horos (https://horosproject.org/). The aneurysm was first identified on 3D TOF MRA images, and then the measurement of morphology was carried out on pre-contrast HR-MRI imaging. In this study, cross-section was defined as the maximal dilation in the cross-sectional plane of FIAs, which is perpendicular to the vessel centerline. The maximal diameter (*D*_max_) of FIAs was defined as the maximum diameter of the cross-section (15). The long-axis section was defined as the plane that covered *D*_max_ and extended in both directions to the sites of 1.5 times the normal diameter in the parent vessel. For more precise positioning of the cross-section and the long-axis section, 3D multiplanar reconstruction (MPR) was adjusted and performed based on different projections (axial, sagittal, and coronal plane) to display the aneurysm. The maximal length (*L*_max_) was defined as the largest diameter of the long-axis section (17). CR_stalk_ was defined as the value of signal intensity (SI) of FIAs' wall divided by SI of the pituitary infundibulum (7). Based on 3D MPR images, the AWE demonstrated by CR_stalk_ was quantitatively measured on the long-axis section and the cross-section, which were manually delineated by one experienced neuroradiologist (more than 20 years in neuroimaging). CR_stalk−long_ demonstrated the mean SI of the aneurysmal wall in the long-axis, which was calculated as ten random points of the aneurysmal wall divided by the mean SI of the stalk on the long-axis section; while on the cross-section, CR_stalk−cross_ was also calculated using the mean SI of the aneurysmal wall divided by the mean SI of the stalk. All MR image analysis was performed by two experienced readers. Discrepancies were resolved through a consensus discussion.

### Statistical analysis

All statistical analyses in this study were conducted by using SPSS (IBM, Armonk, New York, USA). Variables are expressed as mean ± SD. Statistical comparisons were performed by using the Mann–Whitney-*U*-test for continuous variables and χ^2^ test for categorical variables. Univariate analysis was performed by using a non-parametric test (Kruskal–Wallis *H*-test). To identify the risk factors (diameter and CR_stalk_ of FIAs) of symptoms in different measurement methods, we classified the cross-section model, the long-axis model, and the combination model (included parameters in both cross-section model and the long-axis model). *D*_max_, CR_stalk−cross_, and other non-measurement-related parameters were first performed by univariate analysis in the cross-section model. *L*_max_, CR_stalk−long_, and other non-measurement-related parameters were first performed by univariate analysis in the long-axis model, while *D*_max_, CR_stalk−cross_, *L*_max_, CR_stalk−long_ and, other non-measurement-related parameters were first performed by univariate analysis in the combination model. Variables in each model with *P* < 0.20 would be adopted by three models of multivariate logistic regression analysis respectively. *P* < 0.05 was defined as statistical significance. Discrimination, which means the ability to discriminate between symptomatic and symptomatic FIAs, was assessed by the C-statistic (areas under receiver operating characteristics curves, AUC, 0.5 indicates no ability and 1.0 indicates perfect ability). To evaluate the interobserver reliability of CR_stalk_, intraclass correlation coefficient was used.

## Results

**Figure 2:**
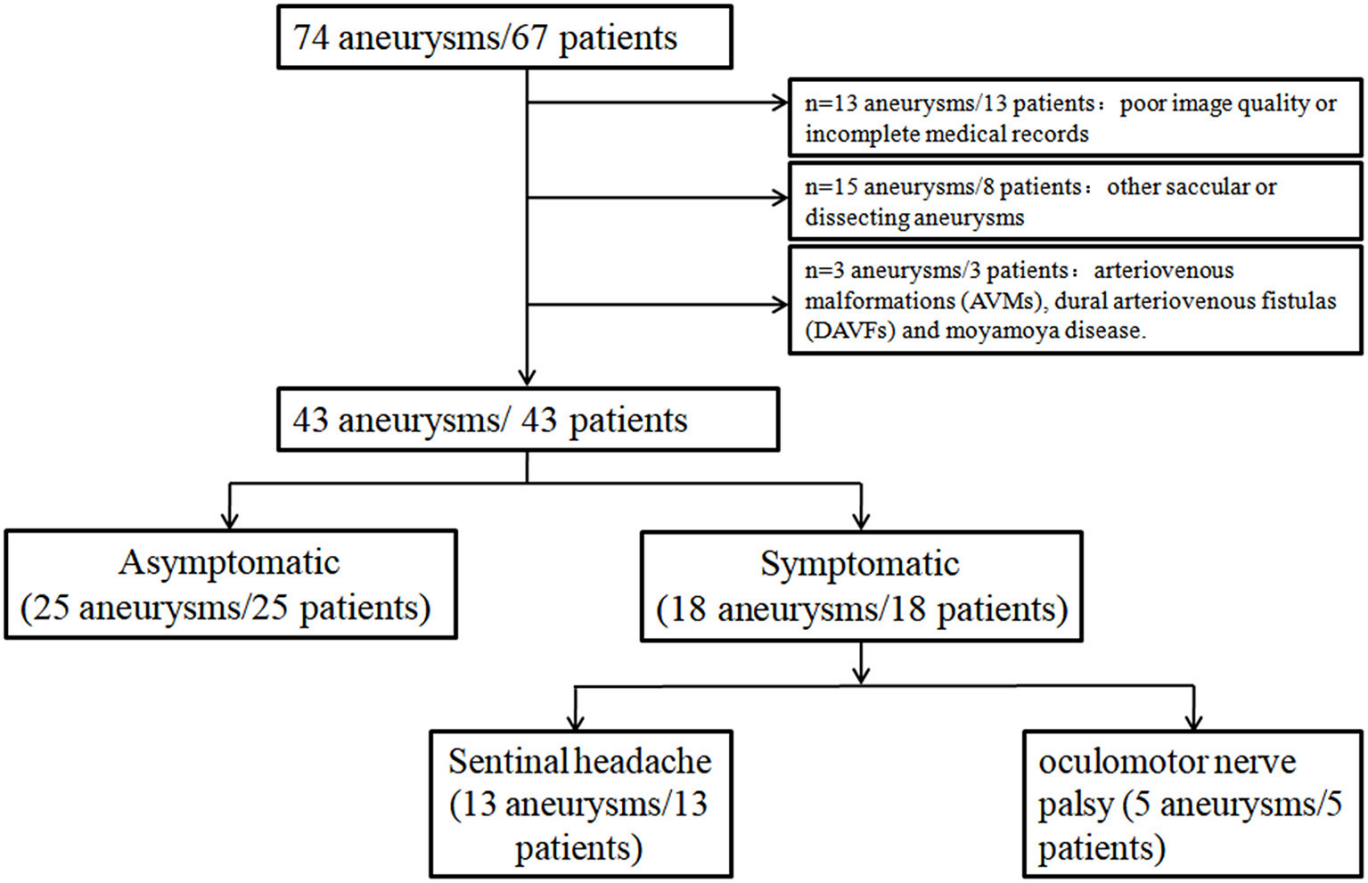
Flowchart of patients' selection.

Thirty-one patients were excluded: 13 with poor image quality or incomplete medical records, 15 were associated with other saccular or dissecting aneurysms, and three were related to AVMs, DAVFs, and moyamoya disease. Finally, a total of 43 patients (mean age was 63.4 ± 12.7 years, male 88.9%) with 43 FIAs were included in this study (Figure 2). Among them, 30 (69.7) were fusiform type, six (14.0) were dolichoectatic type, and seven (16.3) were transitional type. There were 18 (41.9%) patients who presented with aneurysmal symptoms: 13 (30.2) with sentinel headache and five (11.6) with oculomotor nerve palsy. Characteristics of patients and FIAs were listed in Table 1.

**Table 1:** Characteristics of patients and IFAs.

**Characteristics**	
Age, mean ± SD	52.05 ± 10.28
Male, *N* (%)	34 (79.1)
Hypertension, *N* (%)	22 (51.2)
Hyperlipidemia, *N* (%)	14 (32.6)
Diabetes, *N* (%)	8 (18.6)
Current smoker, *N* (%)	8 (58.1)
Location, *N* (%)
ICA	2 (4.7)
BA	20 (46.5)
VA	8 (18.6)
VB	13 (30.2)
Type of IFAs, *N* (%)
Fusiform	30 (69.7)
Dolichoectatic	6 (14.0)
Transitional	7 (16.3)
*D*_max_ (mm)	9.07 ± 2.58
*L*_max_ (mm)	17.72 ± 9.50
Aneurysm symptoms, *N* (%)
Sentinel headache	13 (30.2)
Oculomotor nerve palsy	5 (11.6)
CR_stalk−cross_, mean ± SD	0.88 ± 0.26
CR_stalk−long_, mean ± SD	0.72 ± 0.21

*ICA, internal carotid artery; BA, basilar artery; VA, vertebral artery; VB, vertebral basilar artery; IFAs, intracranial fusiform aneurysms; AWE, aneurysmal wall enhancement; CR_stalk−cross_, the aneurysm-to-pituitary stalk ratio in the cross-section; CR_stalk−long_, the aneurysm-to-pituitary stalk ratio in the long-axis*.

**Figure 3:**
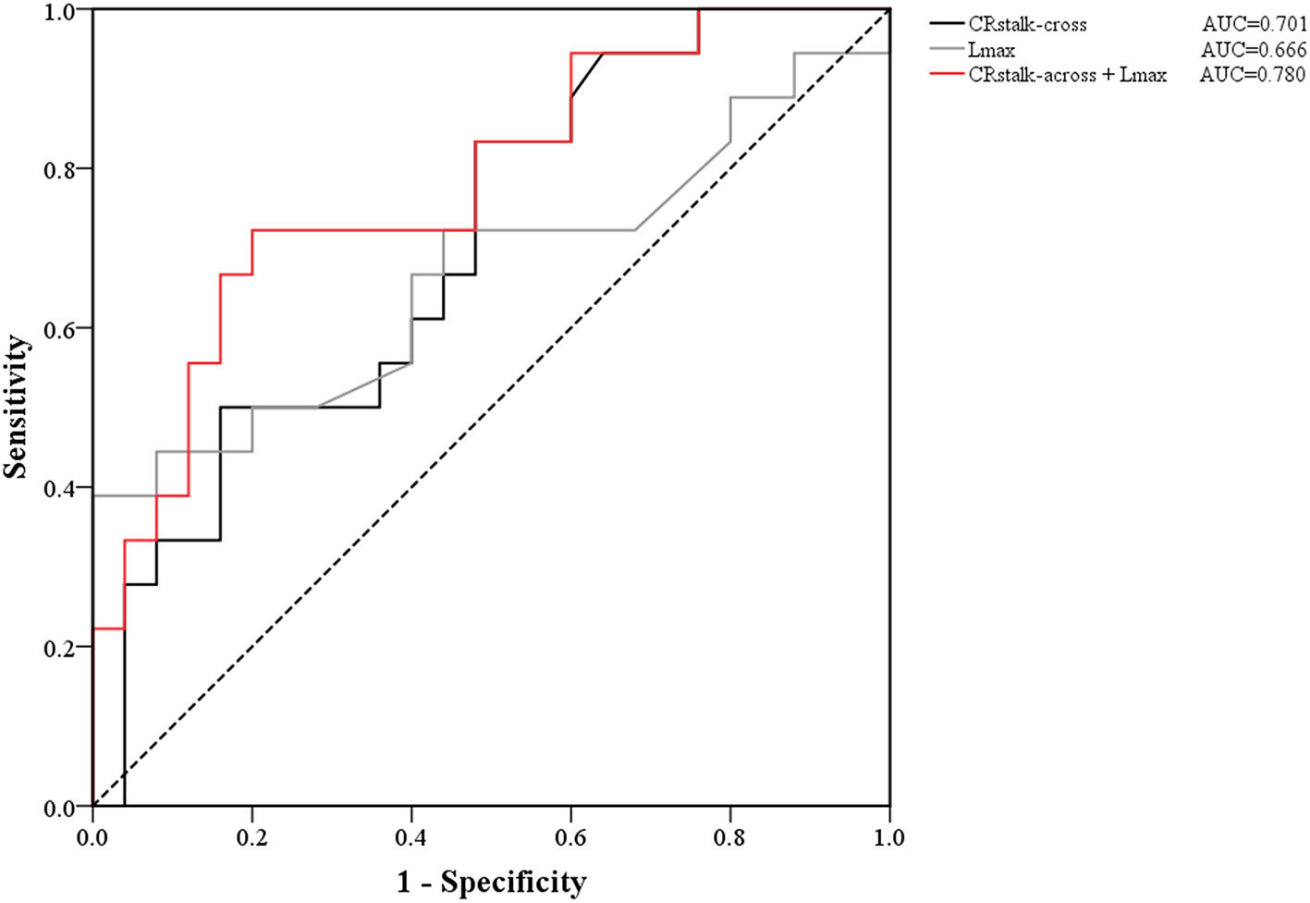
ROC curves of the contrast ratio of CR_stalk−cross_, *L*_max_, and the joint variable (CR_stalk−cross_ + *L*_max_). The AUC value of CR_stalk−cross_, *L*_max_, and CR_stalk−cross_ + *L*_max_ were 0.701, 0.666, and 0.780, respectively. CR_stalk−cross_, aneurysm-to-pituitary stalk in the cross-axis section; *L*_max_, the maximal length of the long-axis section.

In the univariate analysis, the results revealed that male sex (*P* = 0.133), age (*P* = 0.013), FIAs type (*P* = 0.167), mural thrombus (*P* = 0.130), *L*_max_ (*P* = 0.066), CR_stalk−cross_ (*P* = 0.027), and CR_stalk−long_ (*P* = 0.055) tended to be associated with aneurysmal symptoms (Table 2). In the cross-section model of multivariate analysis, male (OR = 7.352, *P* = 0.038), age (OR = 0.914, *P* = 0.018), and CR_stalk−cross_ (OR= 2.346, *P* = 0.048) were independently associated with aneurysmal symptoms. In the long-axis section model of multivariate analysis, male (OR = 7.932, *P* = 0.040), age (OR = 0.900, *P* = 0.010), CR_stalk−long_ (OR = 2.536, *P* = 0.046), and *L*_max_ (OR = 1.138, *P* = 0.019) were independently associated with aneurysmal symptoms. In the combination model of multivariate analysis, male (OR = 9.631, *P* = 0.027), age (OR = 0.900, *P* = 0.011), CR_stalk−cross_ (OR = 2.995, *P* = 0.030), and *L*_max_ (OR = 1.136, *P* = 0.020) were independently associated with aneurysmal symptoms (Table 3). Then we compared the specificity of CR_stalk−cross_ and *L*_max_ in aneurysmal symptoms prediction (Figure 3), and the results revealed that CR_stalk−cross_ has higher accuracy than *L*_max_ (AUC = 0.701 vs. AUC = 0.666) in aneurysmal symptoms prediction, while the combination of CR_stalk−cross_ and *L*_max_ have highest accuracy in aneurysmal symptoms prediction (AUC = 0.780).

**Table 2:** The association between patients' and aneurysmal characteristics and aneurysm symptoms.

**Characteristics**	**Symptomatic FIAs**	**Asymptomatic FIAs**	***P*** **-value**
Male sex, *N* (%)	12 (27.9)	22 (51.2)	0.133
Age, mean ± SD	54.72 ± 10.09	48.33 ± 9.62	0.013^*^
Hypertension	7 (16.3)	15 (34.9)	0.223
Hyperlipidemia	7 (16.3)	7 (16.3)	0.521
Smoking history	10 (23.3)	15 (34.9)	1.000
Aneurysm location			0.646
ICA	0 (0)	2 (4.7)	
BA	9 (20.9)	11 (25.6)	
VA	8 (18.6)	10 (23.3)	
VB	1 (2.3)	2 (4.7)	
IFAs type			0.167
Fusiform, *N* (%)	10 (23.3)	20 (46.5)	
Dolichoectatic, *N* (%)	3 (7.0)	3 (7.0)	
Transitional, *N* (%)	5 (11.6)	2 (4.7)	
*D*_max_	13.25 ± 2.89	8.47 ± 2.79	0.563
*L*_max_	20.65 ± 11.12	9.47 ± 2.39	0.066
Mural thrombus, *N* (%)	11 (25.6)	9 (20.9)	0.130
CR_stalk−cross_	0.96 ± 0.22	0.75 ± 0.28	0.027^*^
CR_stalk−long_	0.79 ± 0.18	0.67 ± 0.21	0.055

*ICA, internal carotid artery; BA, basilar artery; VA, vertebral artery; VB, vertebral basilar artery; FIA, fusiform intracranial aneurysms; CR_stalk−cross_, the aneurysm-to-pituitary stalk ratio in the cross-section; CR_stalk−long_, the aneurysm-to-pituitary stalk ratio in the long-axis section*.

* *P < 0.05*.

**Table 3:** Three models of multivariate logistic regression analysis for risk factors associated with aneurysmal symptoms.

**Multivariate analysis**	**Parameters**	**OR**	**95% CI**	***P*-value**
Cross-section model	Male	7.352	1.113–48.548	0.038^*^
	Age	0.914	0.848–0.984	0.018^*^
	Mural thrombus	2.911	0.559–15.161	0.204
	IFAs type	2.558	0.900–7.271	0.078
	CR_stalk−cross_	2.346	1.006–5.472	0.048^*^
Long-axis section model	Male	7.932	1.100–57.218	0.040^*^
	Age	0.900	0.830–0.975	0.010^*^
	Mural thrombus	1.821	0.286–11.604	0.526
	IFAs type	1.527	0.416–5.607	0.524
	CR_stalk−long_	2.536	1.018–6.323	0.046^*^
	*L*_max_	1.138	1.021–1.269	0.019^*^
Combination model	Male	9.631	1.298–71.437	0.027^*^
	Age	0.900	0.830–0.976	0.011^*^
	IFAs type	1.544	0.426–5.598	0.509
	Mural thrombus	1.312	0.191–9.002	0.783
	CR_stalk−cross_, per SD	2.995	1.115–8.042	0.030^*^
	CR_stalk−long_, per SD	1.054	0.206–5.396	0.949
	*L*_max_	1.136	1.020–1.264	0.020^*^

*CR_stalk−cross_, the aneurysm-to-pituitary stalk ratio in the cross-section; CR_stalk−long_, the aneurysm-to-pituitary stalk ratio in the long-axis; OR, odds ratio*.

* *P < 0.05*.

### Reproducibility of CR_stalk-cross_ and CR_stalk-long_

For the measurement of CR_stalk−cross_ and CR_stalk−long_ from the 43 included patients, the interobserver agreement was excellent [intraclass correlation coefficient = 0.91 (95% CI, 0.87–0.99); 0.88 (95% CI, 0.80–0.96); respectively].

## Discussion

Aneurysmal wall enhancement studies have been widely investigated in SIAs (11, 13, 14, 16). Recently, AWE studies of FIAs have received much attention (2, 7, 15). In the current study, we made the first attempt to compare the accuracy of distinguishing aneurysmal symptoms between parameters that were measured in the cross-section and the long-axis section of FIAs. The results showed that there is an obvious discrepancy in the parameters of the two sections, and CR_stalk−cross_ has the highest specificity in distinguishing aneurysmal symptoms.

Limited studies have investigated the association between AWE parameters and symptoms in FIAs (7, 15). Cao et al. (15) found that in vertebrobasilar nonsaccular aneurysms, enhancement ratio (ER), which corresponds to CR_stalk_ in the current study, has the highest sensitivity and specificity in identifying symptoms than other AWE parameters. In their study, the measurement of AWE was based on the section of the aneurysmal wall with the highest signal intensity. In another recent study, the authors defined CR_stalk_ > 0.60 as enhancement. The results demonstrated that headache was associated with fusiform aneurysm enhancement (7). In their study, the measurement of AWE was based on the section at the maximal diameter, while in our study, we chose both the cross-section at the maximal diameter and the long-axis section along with the superimposed dilation of the involved artery and compared CR_stalk_ in these two sections. Among all the parameters included in this study, CR_stalk_ in the cross-section (CR_stalk−cross_) has the highest performance in predicting aneurysmal symptoms. Notably, a recent study showed that AWE of the parent vessel 3 mm from the neck was higher than 5 mm from the neck (7), and since the aneurysm neck of FIAs is often not obvious due to the long dilation of the involved artery, the extent of AWE in FIAs may also be increased when closer to the most dilated areas. Similarly, the signal intensity of AWE in the long-axis section may tend to be heterogeneous and lower than in the cross-section of the most obvious dilation. Therefore, we compared the value of CR_stalk_ between the two sections, and the mean value of CR_stalk_ is higher in the cross-section than in the long-axis section (0.88 vs. 0.73, respectively, *P* < 0.001), which confirmed our hypothesis. Many previous studies have demonstrated that AWE represents aneurysmal wall IPs (10, 11), which were reported to play a key role in aneurysm growth and rupture (9, 18). As we mentioned above, CR_stalk_ is higher in the cross-section than in the long-axis section. Thus, the extent of IPs of the aneurysmal wall in the cross-section with the maximal diameter may be higher than in the long-axis section. Therefore, we propose that for FIAs, the area with local higher AWE (cross-section with the maximal diameter) may be more inclined to grow, which needs further verification in future longitudinal studies.

In the long-axis section model, CR_stalk−long_ and *L*_max_ were independent predictors of aneurysm symptoms, which revealed that AWE in the long-axis section can also predict aneurysmal symptoms. Further, compared with the cross-section, which only demonstrates the section with the maximum expansion in FIAs, the long-axis section demonstrates the section along the FIAs and the parent vessel. Therefore, CR_stalk_ in the long-axis section (CR_stalk−long_) tends to exhibit the Ips' burden on the entire FIA, while CR_stalk−cross_ demonstrates that the section, which is transverse to the aneurysm trunk with the most obvious expansion, may exhibit the area with the highest Ips' burden. Still, both the two sections were performed in 2D views of FIAs, so it would be better to obtain the whole IPs burden on the FIA by using new techniques (e.g. 3D space) in future studies.

Previous studies revealed that *D*_max_ was also related to FIAs-related symptoms (15). However, *D*_max_ was not associated with aneurysmal symptoms in this study, and in contrast, *L*_max_ was the independent predictor of aneurysmal symptoms (*P* = 0.008). Generally, symptoms were often due to the compression caused by the mass effect of bigger aneurysms (19). *L*_max_ demonstrates the length of the longitudinal section of FIAs and the superimposed dilation of the involved artery, which may better reflect the mass effect of fusiform aneurysms on the brain tissue than *D*_max_. In the cross-section model, CR_stalk−cross_ was the independent predictor of aneurysmal symptoms (*P* = 0.048). We also found that the performance for discriminating aneurysmal symptoms was improved when combining CR_stalk−cross_ and *L*_max_ (Figure 3). Therefore, in the future, CR_stalk_ in the cross-section and the length of the long-axis section could be combined to improve the predictive performance of aneurysmal symptoms. Considering aneurysmal symptoms may indicate instability of IAs (14), those risk factors of aneurysmal symptoms (e.g. CR_stalk_, *L*_max_, and their combination) may help to stratify aneurysm rupture risk.

Other risk factors associated with symptoms include IFA types and mural thrombus (15, 20). The results of these two risk factors did not reach statistical significance. We considered it due to the limited sample size in this study.

### Strengthens and limitations

This is the first study to make a quantitative evaluation of AWE in two sections for discriminating FIAs' symptoms based on HR-MRI. However, there are several inherent limitations in the current study. First, the overall sample size was small. Additionally, the limited sample size in dolichoectatic and transitional groups restricted the sub-group analysis for the three IFA types, which should be carried out in a larger cohort in future studies. Second, HR-MRI images were exported by three different 3T MRI machines (GE, Simens, and Philip) although the inherent parameters were adjusted to be consistent. Third, flow artifacts can mimic aneurysm wall enhancement in FIAs. In future studies, advanced blood suppression techniques like MSDE (motion-sensitized driven-equilibrium) (21) and DANTE (delay alternating with nutation for tailored excitation preparation module) (22) are needed to help to distinguish artifacts and improve the characterization of AWE. Fourth, the authors concentrated on two different sections which maybe not sufficiently manifest the overall AWE conditions of FIAs. More accurate methods like 3D space technology should be carried out in the future (23).

## Conclusion

Aneurysm wall inflammation which can be demonstrated as enhancement is highly suggestive of inflammation, but not always. AWE on the cross-section of FIAs with the most obvious expansion may be located in higher aneurysmal IPs. CR_stalk−cross_ and *L*_max_ were independent risk factors for aneurysmal symptoms. The combination of these two factors may improve the predictive performance of aneurysmal symptoms and may also help to stratify the instability of FIAs.

## Data availability statement

The raw data supporting the conclusions of this article will be made available by the authors, without undue reservation.

## Ethics statement

The studies involving human participants were reviewed and approved by Institutional Review Board of Beijing Tiantan Hospital. The patients/participants provided their written informed consent to participate in this study.

## Author contributions

FP made the article writing. LL made the conception and design of the study. XF, HN, HZ, XB, ZL, XH, JX, and BX made the acquisition, analysis, and interpretation of the data. BS and AL made the critical revision of the article. All authors contributed to the article and approved the submitted version.

## Funding

This work was supported by the Natural Science Foundation of China (82171290 and 81771233), the Natural Science Foundation of Beijing, China (L192013, 22G10396, and 7142032), and Beijing Municipal Administration of Hospitals' Ascent Plan (DFL20190501).

## Conflict of interest

The authors declare that the research was conducted in the absence of any commercial or financial relationships that could be construed as a potential conflict of interest.

## Publisher's note

All claims expressed in this article are solely those of the authors and do not necessarily represent those of their affiliated organizations, or those of the publisher, the editors and the reviewers. Any product that may be evaluated in this article, or claim that may be made by its manufacturer, is not guaranteed or endorsed by the publisher.
